# A phase-locked loop epilepsy network emulator for localizing, forecasting, and controlling ictal activity

**DOI:** 10.1186/1471-2202-16-S1-P38

**Published:** 2015-12-18

**Authors:** Patrick D Watson, Kevin Horecka, Rama Ratnam, Neal J Cohen

**Affiliations:** 1 Beckman Institute of Science and Technology, UIUC, IL, USA; 2 Neuroscience Program, UIUC, IL, USA; 3 Department of Psychology, UIUC, IL, USA; 4 Coordinated Science Laboratory, UIUC, Urbana, IL, USA; 5 Advanced Digital Sciences Center, Illinois at Singapore Pte. Ltd., Singapore

## 

Seizure detection is rapidly improving thanks to novel computational approaches [1] and public databases of electrocorticography (ECoG) data [2]. But these approaches rarely model the high-frequency oscillations that underlie seizure pathology [3]. In the current work, we employ a phase-locked loop (PLL) neural network [4] to emulate a mesoscale circuit undergoing high-frequency oscillation. We phase-lock the nodes of the emulator to raw voltages recorded from chronically implanted ECoG electrodes in a canine model of epilepsy, and demonstrate that the emulator experiences uncontrolled phase oscillation far in advance of either behaviorally observed seizure activity or fluctuations in ECoG voltages. Using distance weighting to train the epilepsy network emulator, we localize the ECoG electrodes responsible for destabilization, and present measures sensitive to these phase disruptions to establish a forecasting period for ictal activity. We discuss how phase oscillations from real-time epilepsy network emulation could serve as a closed-loop feedback control signal to interrupt ictal activity.
